# PharmDB-K: Integrated Bio-Pharmacological Network Database for Traditional Korean Medicine

**DOI:** 10.1371/journal.pone.0142624

**Published:** 2015-11-10

**Authors:** Ji-Hyun Lee, Kyoung Mii Park, Dong-Jin Han, Nam Young Bang, Do-Hee Kim, Hyeongjin Na, Semi Lim, Tae Bum Kim, Dae Gyu Kim, Hyun-Jung Kim, Yeonseok Chung, Sang Hyun Sung, Young-Joon Surh, Sunghoon Kim, Byung Woo Han

**Affiliations:** 1 Medicinal Bioconvergence Research Center, Seoul National University, Seoul 152–742, Republic of Korea; 2 Research Institute of Pharmaceutical Sciences, College of Pharmacy, Seoul National University, Seoul 151–742, Republic of Korea; 3 Information Center for Bio-pharmacological Network, Seoul National University, Suwon 443–270, Republic of Korea; 4 Department of Molecular Medicine and Biopharmaceutical Sciences, Seoul National University, Seoul 151–742, Republic of Korea; 5 College of Pharmacy, Chung-Ang University, Seoul 156–756, Republic of Korea; Wayne State University School of Medicine, UNITED STATES

## Abstract

Despite the growing attention given to Traditional Medicine (TM) worldwide, there is no well-known, publicly available, integrated bio-pharmacological Traditional Korean Medicine (TKM) database for researchers in drug discovery. In this study, we have constructed PharmDB-K, which offers comprehensive information relating to TKM-associated drugs (compound), disease indication, and protein relationships. To explore the underlying molecular interaction of TKM, we integrated fourteen different databases, six Pharmacopoeias, and literature, and established a massive bio-pharmacological network for TKM and experimentally validated some cases predicted from the PharmDB-K analyses. Currently, PharmDB-K contains information about 262 TKMs, 7,815 drugs, 3,721 diseases, 32,373 proteins, and 1,887 side effects. One of the unique sets of information in PharmDB-K includes 400 indicator compounds used for standardization of herbal medicine. Furthermore, we are operating PharmDB-K via phExplorer (a network visualization software) and BioMart (a data federation framework) for convenient search and analysis of the TKM network. Database URL: http://pharmdb-k.org, http://biomart.i-pharm.org.

## Introduction

It is known that Traditional Medicine (TM) originated in China about 3,000 years ago, and was introduced to Korea in the 6^th^ century [1]. Although TM began in China, it has been indigenized in Korea and developed into the unique Traditional Korean Medicine (TKM). In Korea, the TKM hospital industry continues to grow, and annual revenue of the TKM industry is expected to increase to $5.8 billion in 2015 [2]. However, TKM has not been well recognized worldwide thus far. Since TM has been brought to the attention of pharmaceutical companies for novel lead compounds, some databases for Traditional Chinese Medicine (TCM) have been developed and widely used [3–9]. Unfortunately, however, there is no well-known integrated bio-pharmacological TKM database for drug development.

Traditional herbal medicines consist of unpurified plant extracts or portions of plants containing several compounds. The efficacy of traditional herbal medicine depends on the compounds that it contains. Therefore, correct identification of indicator compounds in specific herbs is an essential prerequisite to relating their medical benefits with known disease indicators. As mentioned earlier, there are a number of well-known TCM databases. Traditional Chinese Medicines Information Database (TCM-ID) contains information for prescriptions, herbs, and ingredients, but there is no information about related proteins [3]. TCM@Taiwan contains information on a large number of compounds isolated from herbs, but information on related diseases and proteins is missing [4]. Traditional Chinese Medicines Integrated Database (TCMID) is the first database containing comprehensive information on interactions between compounds, proteins, and herbs, and it is likely the largest database in related fields [5]. However, since TCMID uses STICH, a resource containing known and predicted interactions from diverse organisms [10], for compound-protein interaction data without any critical filtration, it is possible that TCMID may contain unnecessary compound-protein data for drug discovery. There are also many other TCM databases such as Traditional Chinese Medicine Systems Pharmacology Database (TCMSP), TCMGeneDIT, China Natural Products Database (CNPD), and Comprehensive Herbal Medicine Information System for Cancer (CHMIS-C) [6–9]. These TCM databases provide diverse types of information including hundreds of compounds (ingredients) for each herb. However, due to a lack of detailed information, it is difficult to recognize which compounds play major roles as indicators or active compounds.

As mentioned earlier, although TKM was started based on TCM, TKM has been developed to a unique medicinal category by acquiring region-specific medical experiences for hundreds of years. We compared our TKM-disease relationship data with TCM-ID to see the differences between TKM and TCM and found them to be not quite identical. For example, skin diseases are commonly found in two databases for indication of Isatidis Folium. Moreover, hepatitis A, hepatitis B, cholecystitis, and cholelithiasis are listed in PharmDB-K, but not in TCM-ID. Allii Bulbus has been used for anthelmintic, toxication reduction, and itching in China. However, in Korea, there are other distinct reasons for Allii Bulbus use, such as cold, snake bites, diarrhea, edema, and pain. These results suggest that TKM provides other new potentials of herbs that are not covered by TCM.

Due to the reasons mentioned above, we have developed PharmDB-K, an integrated bio-pharmacological network database for TKM. PharmDB-K has three unique strengths: 1) it is an integrated TKM-Drug-Protein-Disease network; 2) it contains manually curated information about indicator compounds for herbs; 3) it has diverse tools for analysis.

## Implementation

### Integrated TKM-Drug-Protein-Disease network

**Fig 1:**
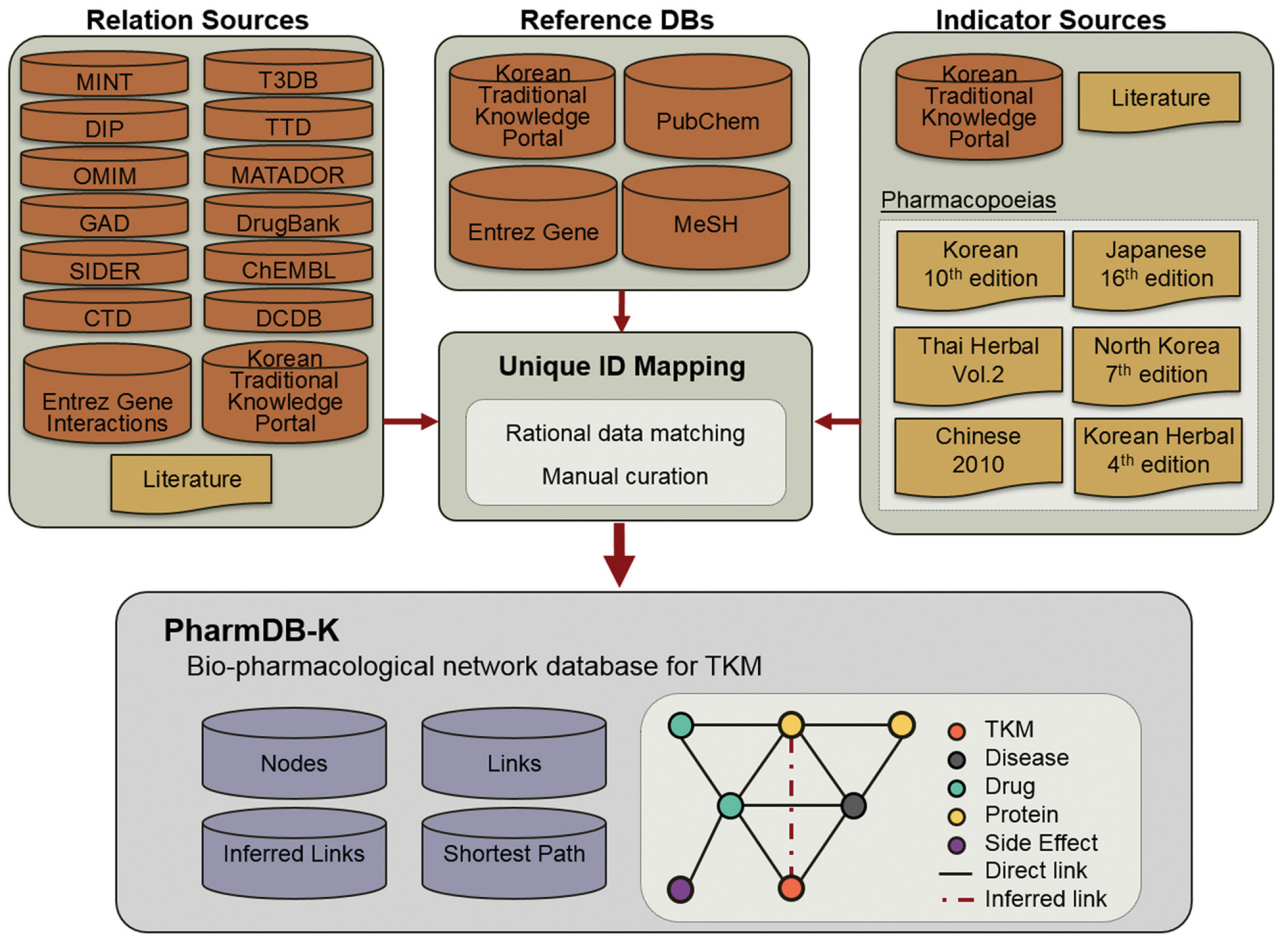
Overview of PharmDB-K. Fourteen databases, six pharmacopoeias, and literature were integrated using four different reference databases to build PharmDB-K.

**Fig 2:**
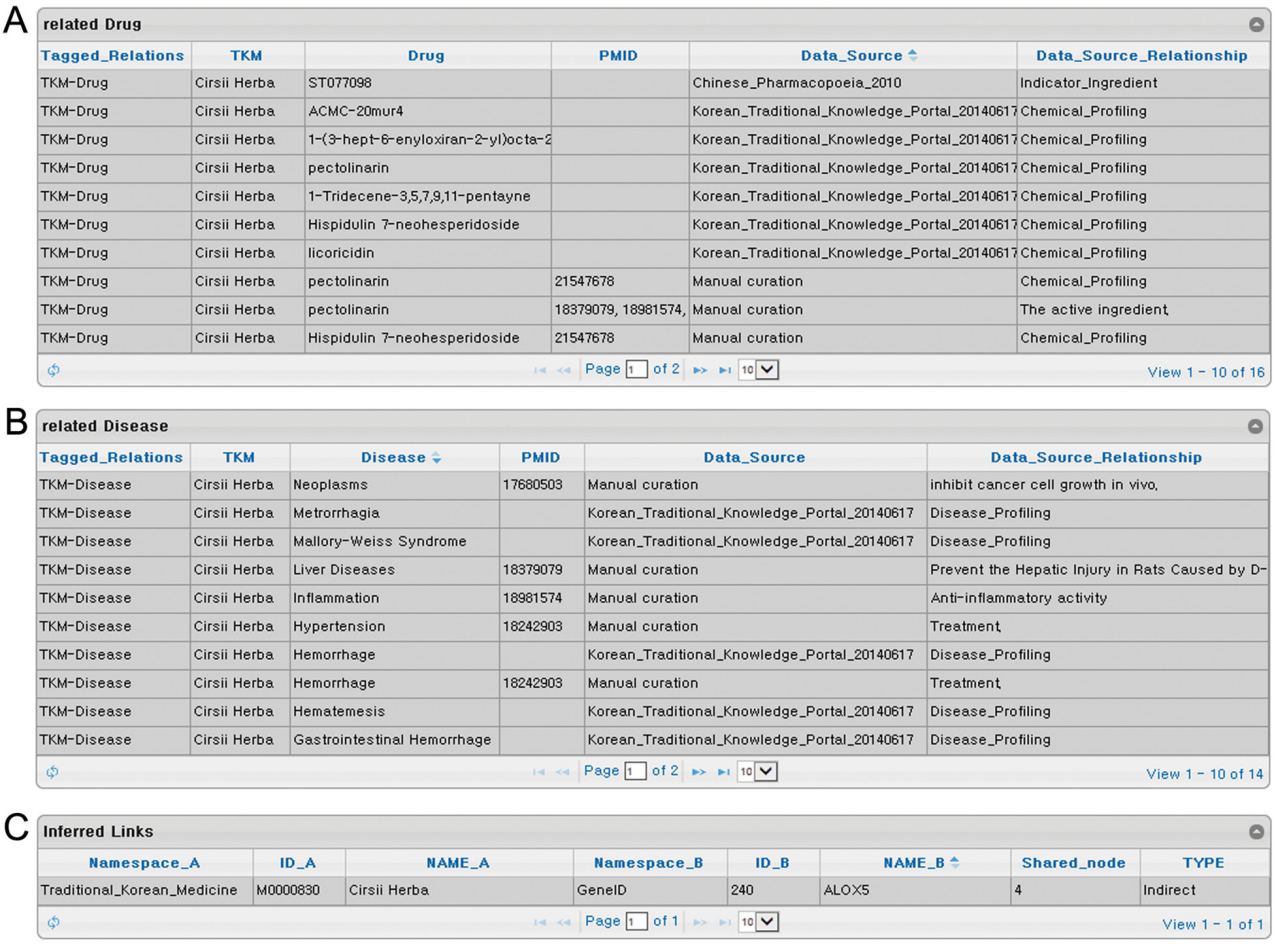
Detailed relational data on TKM. (A) Known TKM-Drug relation data. (B) Known TKM-Disease relation data. (C) Inferred TKM-Protein relation data.

Although the number of articles about TKM has been increasing, most research has focused on profiling chemicals. So, scientific knowledge for analyzing and uncovering the mechanisms of actions is still insufficient. In order to overcome this limitation, fourteen different databases (ChEMBL, CTD, DCDB, DIP, DrugBank, Entrez Gene Interactions, GAD, MATADOR, MINT, OMIM, SIDER, T3DB, Traditional Knowledge Portal, and TTD), six pharmacopoeias, and published articles were integrated to build a bio-pharmacological network that connects compounds found in herbs to known drugs, diseases, proteins, and side effects (Fig 1, S1 Table) [11–23]. For data integration in a unified format, we adopted PubChem CID for drugs, Entrez Gene ID for proteins, MeSH descriptor for diseases and side effects, and Med CD number of Korean Traditional Knowledge Portal for TKMs.[24–27] PharmDB-K consists of five kinds of nodes: TKMs, drugs, diseases, proteins, and side effects. And it is composed of eight different relationship categories: TKM-Disease, TKM-Drug, Drug-Disease, Drug-Drug, Drug-Protein, Drug-Side Effect, Disease-Protein, and Protein-Protein (Table 1). We categorized FDA approved drugs and all types of compounds including experimental compounds, indicator compounds, and ingredient compounds of herbs into the Drug node because of their potential as a new drugs. So, the TKM-Drug relationship primarily explains profiles of indicator compounds, active compounds, and chemicals from herbs (Fig 2A).

**Table 1:** Data resources.

Category	Data sources	Number of relationships
TKM-Disease	Korean Traditional Knowledge Portal, Literature	2,184
TKM-Drug	Chinese Pharmacopoeia 2010, Japanese Pharmacopoeia 16^th^ Edition, Korean Herbal Pharmacopeia 4^th^ Edition, Korean Pharmacopoeia 10^th^ Edition, North Korea Pharmacopoeia 7^th^ Edition, Thai Herbal Pharmacopoeia vol.2, Korean Traditional Knowledge Portal, Literature	5,087
Drug-Disease	CTD, DCDB, TTD, Literature	55,874
Drug-Drug	DCDB, DrugBank, Literature	21,956
Drug-Protein	ChEMBL, CTD, DCDB, DrugBank, MATADOR, TTD, T3DB, Literature	130,617
Drug-Side Effect	SIDER	80,229
Disease-Protein	CTD, GAD, OMIM, TTD	161,292
Protein-Protein	DIP, Entrez Gene Interactions, MINT	158,886

Since TKM has been developed for over a thousand years, indications for the use of TKM are described as either old disease names or symptoms that do not exactly match modern medicinal terms. This has become a big obstacle in utilizing TKM for modern drug development. To overcome this problem, we converted these symptoms and disease terms in TKM into MeSH descriptors [27]. TKM-Disease relationship information was imported from Korean Traditional Knowledge Portal that originated from traditional Korean medical books, Donguibogam (published in 1613) and Ungokbonchohak (published in 2004), and literature (Fig 2B) [24]. At present, PharmDB-K contains 342 MeSH descriptors for herbs and 3,721 MeSH descriptors in total.

Although PharmDB-K is the first integrated network database for TKM, it is not the first and biggest database for herbs. However, PharmDB-K has some unique strengths. PharmDB-K integrates seven different databases along with literature, and predicted data have been eliminated to collect only verified compound-protein interaction data. Furthermore, the compounds isolated from herbs were manually curated and converted into PubChem CIDs based on their names and structures [26]. Thereafter, PubChem CID has been used for compound-associated data integration to avoid mismatch problems. Taken together, we believe that PharmDB-K contains a relatively small but more reliable Drug-Protein data set, and it can also provide inferred TKM-Protein links for further research (Fig 2C).

### Indicator compounds: manually curated key compounds

A pharmacopoeia is a book containing information about standards and quality specifications for medicines and is published by a national or regional authority. In certain Asian countries, pharmacopoeias also contain indicator compound information for herbal medicines. The indicator compound information is used to identify and confirm medicinal performance characteristics. Additionally, they can provide valuable information for establishing solid connections between herbal medicines and modern medicinal chemistry. This indicator compound information has been collected from pharmacopoeias of five Asian countries: China, Japan, South Korea, North Korea, and Thailand [28–34]. The herbs that do not have indicator compounds in these pharmacopoeias were excluded from PharmDB-K. So, PharmDB-K currently contains 250 herbs that have indicator compounds. PharmDB-K contains more than 400 indicator compounds and about 5,000 compounds isolated from herbs. The chemical information was manually curated, and chemicals without available PubChem CIDs were ignored. Additionally, compounds known (or expected) to have medicinal benefits are referred to as “active ingredients”, and these data were acquired from the literature.

**Fig 3:**
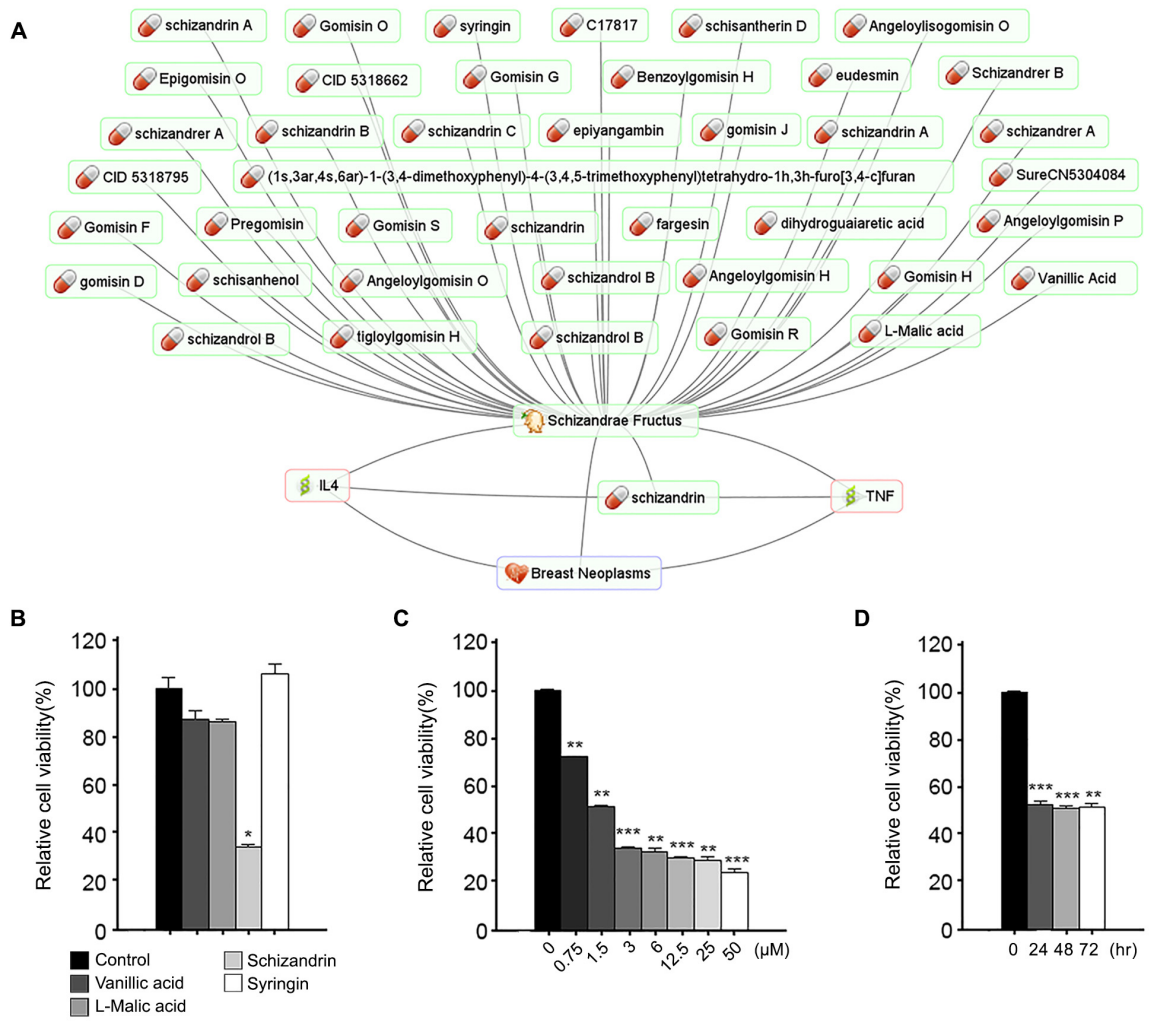
Role of schizandrin in Schizandrae Fructus. (A) Schizandrae Fructus-centered network. (B-D) Selected 4 chemicals (50 μM) were treated for 72 hr. Chemical-treated MDA-MB-231 cells were subjected to MTT assay to check the cell viability (B). Schizandrin were treated dose (C) and time (D) dependently as indicated, and cell viability was checked as above. DMSO were used as a control. The experiments were repeated three times. The error bar means S.D. *p<0.05; **<p0.01; ***p<0.001.

Indicator compounds work as major active compounds in some cases. Schizandrin is one of the main dibenzocyclooctadiene lignans present in Schizandrae Fructus (Fig 3A). According to Korean and Thai Pharmacopoeias, schizandrin is an indicator compound for Schizandrae Fructus. It has been demonstrated that schizandrin reduces protein levels of TNF-alpha and IL-4 and exhibits growth inhibition effect on human breast cancer cell lines [35, 36]. We evaluated the antitumor effect of schizandrin compared with three randomly selected compounds exit in Schizandrae Fructus. Among them, only schizandrin significantly suppressed the cell viability in breast cancer cells (Fig 3B). As shown in Fig 3C and 3D, the cell viability was reduced by schizandrin in a dose- and time-dependent manner. Collectively, these data suggest that schizandrin is likely the active compound of Schizandrae Fructus as an antitumor agent.

**Fig 4:**
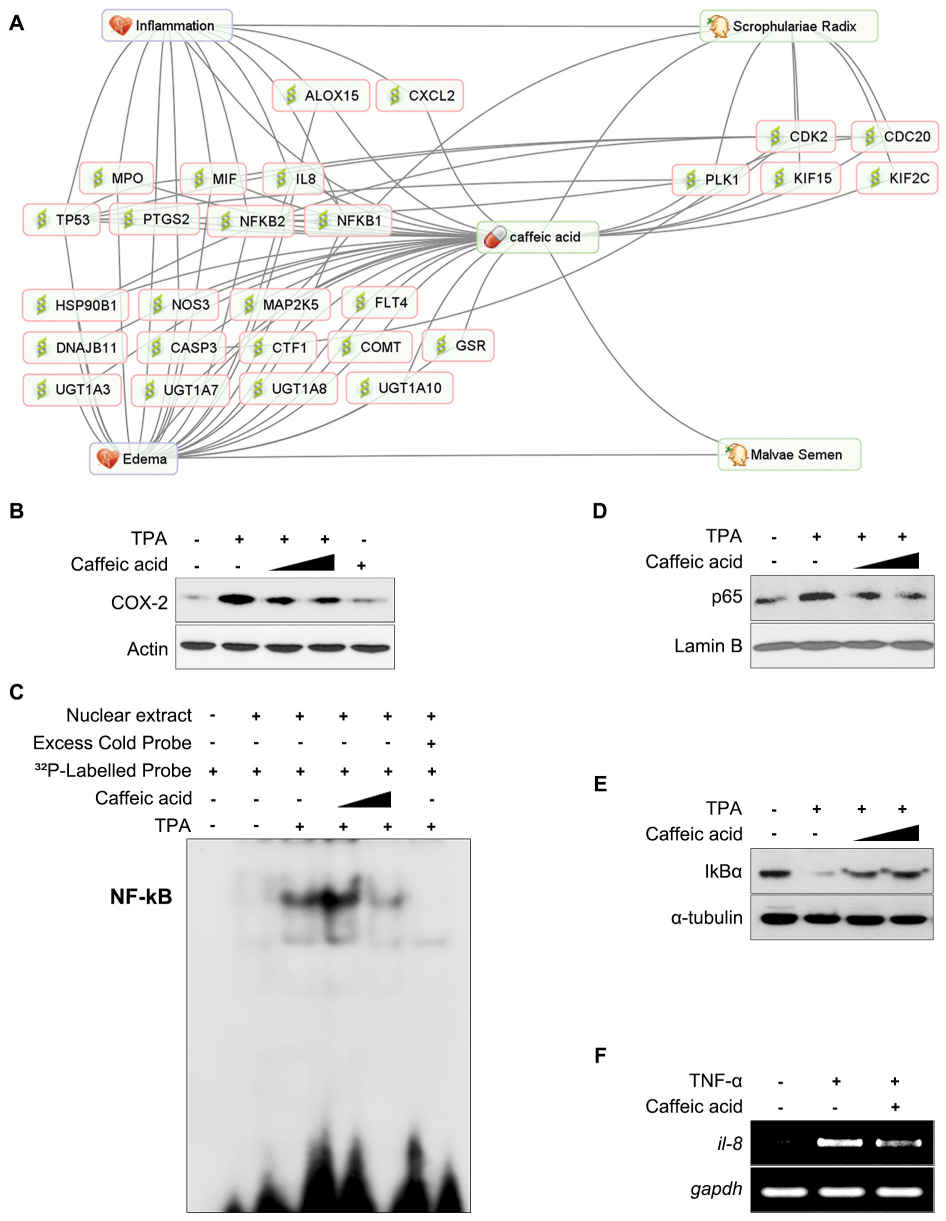
Caffeic acid attenuates the expression of COX-2 and IL-8 as well as NF-κB activation in HaCaT cells. (A) Possible effect of caffeic acid in Scrophulariae Radix and Malvae Semen. (B) HaCaT cells were pretreated with caffeic acid (50 and 100 μM) for 1 hr, and then cells were exposed to TPA (100 nM) for additional 8 hr. (C) Cells were treated with TPA (100 nM) in the presence of caffeic acid (50 and 100 μM) for 2 hr. The NF-κB DNA binding activity was assessed by the gel-shift assay. The nuclear extracts were prepared and incubated with the radiolabeled oligonucleotides containing κB consensus sequence for the analysis of NF-κB DNA binding by EMSA. (D) Nuclear proteins were separated by 10% SDS-polyacrylamide gel electrophoresis and immunoblotted with p65 antibody. Lamin B was used as markers of nuclear proteins. (E) The cytosolic extracts prepared from cells incubated with TPA for 3 hr in the presence or absence of caffeic acid were immunoblotted with was analyzed by Western blotting to examine the expression of IκBα. (F) HaCaT cells were treated with TNF-α (20 nM) in the absence or presence of caffeic acid (100 μM) for 24 hr and then the isolated RNA was reverse-transcribed and amplified as described in Materials and Methods. Expression of il-8 and gapdh mRNA was measured by RT-PCR.

Fig 4A illustrates another interesting example regarding roots of Scrophulariae Radix, which are used as an anti-inflammatory agent [37]. It was reported that caffeic acid is one of active compounds in Scrophulariae Radix and has an anti-inflammatory effect by suppressing NF-kB and COX-2 (PTGS2) [38, 39]. According to Chinese Pharmacopoeia, caffeic acid is also an indicator compound for Malvae Semen. Although Malvae Semen is used in the treatment of edema in South Korea, its mechanism of action is still unknown [24]. The therapeutic effect of caffeic acid on edema has already been demonstrated, and there are a number of shared proteins between caffeic acid and Malvae Semen including NFKB1, NFKB2, and PTGS2 [38]. It is possible, therefore, that caffeic acid could be the active compound in Malvae Semen. To evaluate the effect of caffeic acid on edema, we investigated the role of caffeic acid in immune system. Tumor-promoting activity of 12-O-tetradecanoylphorbol-13-acetate (TPA) induces skin edema, epidermal hyperplasia and inflammation [40]. Pretreatment of HaCaT cells (a human keratinocyte cell line) with caffeic acid attenuated TPA-induced expression of COX-2 protein in a concentration-dependent manner (Fig 4B). The expression of COX-2 is transcriptionally regulated by several transcription factors including NF-κB. We examined the effects of caffeic acid on TPA-induced activation of NF-κB in HaCaT cells. Caffeic acid treatment significantly inhibited TPA-induced DNA binding of NF-κB and nuclear translocation of its active subunit of p65/RelA (Fig 4C and 4D). In addition, caffeic acid inhibited the subsequent degradation of IκBα in TPA-stimulated HaCaT cells (Fig 4E). Moreover, as shown in Fig 4F, the upregulation of il-8 (interleukin 8) mRNA transcript by tumor necrosis factor-α (TNF-α) was significantly reduced by caffeic acid in HaCaT cells. In summary, caffeic acid inhibited the activation of NF-κB which is a major transcription factor involved in the regulation of COX-2 expression in TPA-treated HaCaT cells. In addition, we also observed that caffeic acid blocked the expression of interleukin-8, cytokine considered to play a role under inflammatory situation. Therefore, these findings support our hypothesis that caffeic acid could be the major active compound of Malvae Semen for treatment of edema. These data suggest that PharmDB-K is a useful resource for narrowing down and predicting active compounds among compounds found in herbs and for establishing hypotheses on the functional mechanisms of herbs.

### Inferred protein links for TKM

**Fig 5:**
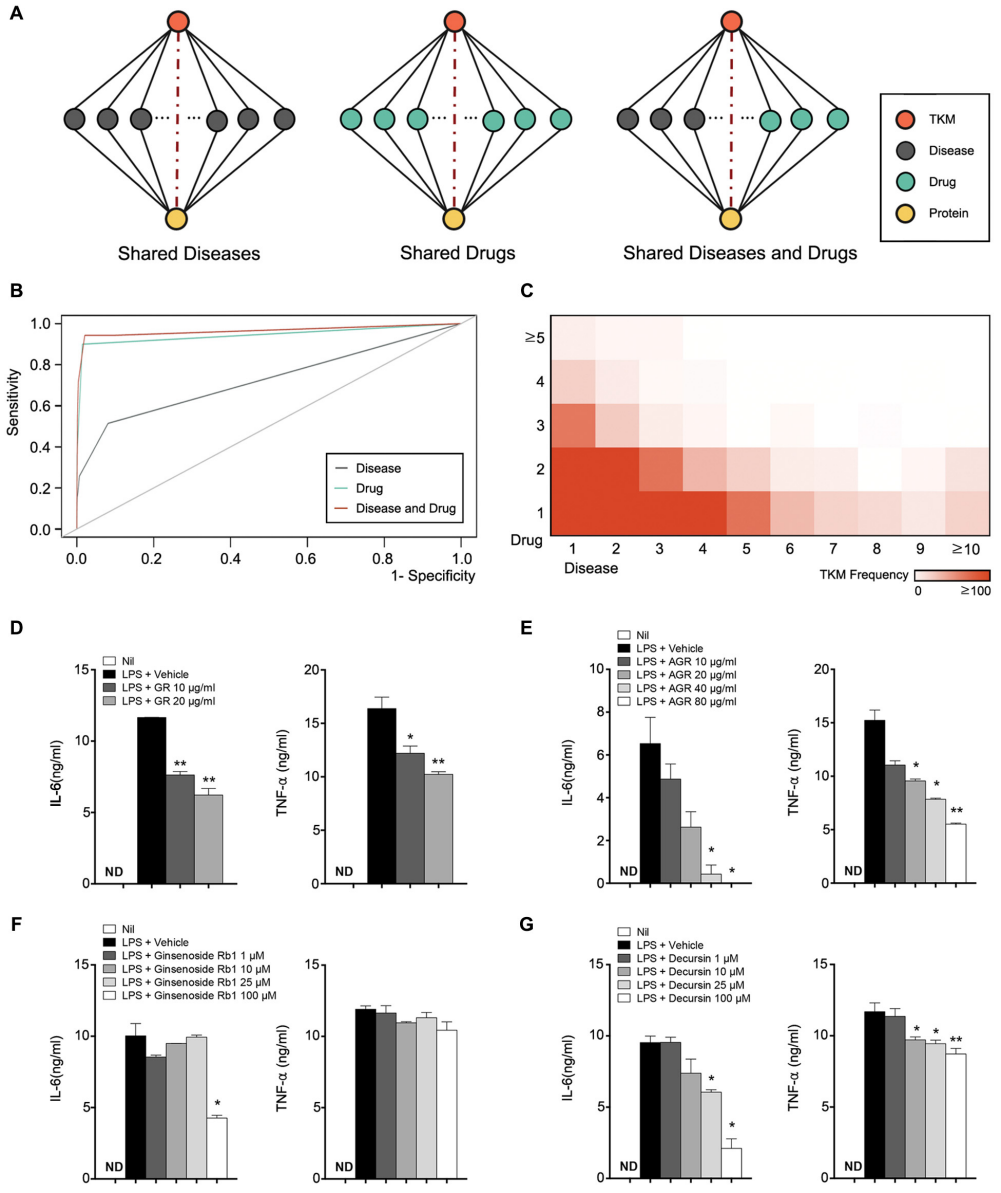
Inferred TKM-Protein relation. (A) Basic idea of inferred TKM-Protein relation. The probability of connection between TKM and Protein increases as the “Shared node count” increases. (B) ROC analysis of inferred TKM-Protein relationships using disease only, drug only, and both. 16 TKMs with known TKM-Protein relationships were used for this analysis. (C) Shared node count frequency. (D-G) Effects of extract and compound of Ginseng Radix and Angelicae Gigantis Radix on the expression of IL-6 and TNF-α upon LPS stimulation. Raw264.7 cells were stimulated with LPS (100 ng/ml) together with either Ginseng Radix (GR), Angelicae Gigantis Radix (AGR) extract, ginsenoside rb1 or decursin at indicated concentration for 24 hr. The amounts of IL-6 and TNF-α produced in the cultured supernatants were measured by ELISA. Data shown are mean ± SEM. *p<0.05; **<p0.01; ***p<0.001; ND, not detected.

Previously, we developed the Shared Neighborhood Scoring (SNS) algorithm to generate inferred links [41]. Unfortunately, however, the SNS algorithm could not be applied to TKM since there was only a limited amount of known data regarding the TKM-Protein relationship. The probability of a connection between two nodes showed monotonic increase with “Shared nodes count” in PharmDB [41]. Based on this observation, inferred TKM-Protein relational data were generated using the number of shared nodes between them (Fig 5A). The numbers of inferred TKM-Protein relationships were 200,481, 123,382, and 7,501, based on “shared Diseases count”, “shared Drugs count”, and “shared Diseases and Drugs count”, respectively. We collected known TKM-Protein relation data for 16 TKMs from the literature and they were used to validate the inferred links. The result was measured by ROC curves (Fig 5B). For “shared Diseases and Drugs count” cases, “Drugs count” was assigned with a weight of 2. AUC values for “shared Diseases count”, “shared Drugs count”, and “shared Diseases and Drugs count” were 0.726, 0.945, and 0.965, respectively. Among the inferred TKM-Protein relation based on “shared Diseases and Drugs count”, a total of 7,501 relations shared at least two nodes from two different categories (e.g., one from Disease and one from Drug) (Fig 5C). And these types of relations were used as final inferred protein links for TKMs.

PharmDB-K predicted that Ginseng Radix and Angelicae Gigantis Radix may regulate the production of IL-6 and TNF-α, pro-inflammatory cytokines that are produced by macrophages for both innate and adaptive immunity. To validate this inferred TKM-Protein relation, Raw264.7 cells were stimulated with LPS in the presence of increasing doses of extracts from Ginseng Radix and Angelicae Gigantis Radix, ginsenoside Rb1 (an indicator compound for Ginseng Radix), and decursin (an indicator compound for Angelicae Gigantis Radix). The levels of LPS-induced IL-6 and TNF-α were all significantly decreased by the addition of Ginseng Radix extracts, Angelicae Gigantis Radix extracts, and decursin in a dose-dependent manner (Fig 5D, 5E and 5G). Addition of ginsenoside Rb1 also inhibited the production of IL-6, but the production of TNF-α was not affected by the same treatment (Fig 5F). These results demonstrate that Ginseng Radix, Angelicae Gigantis Radix, and their indicator compounds, ginsenoside Rb1 and decursin, regulate the production of IL-6 and TNF-α from macrophages as PharmDB-K inferred.

### Tools: phExplorer and BioMart

**Fig 6:**
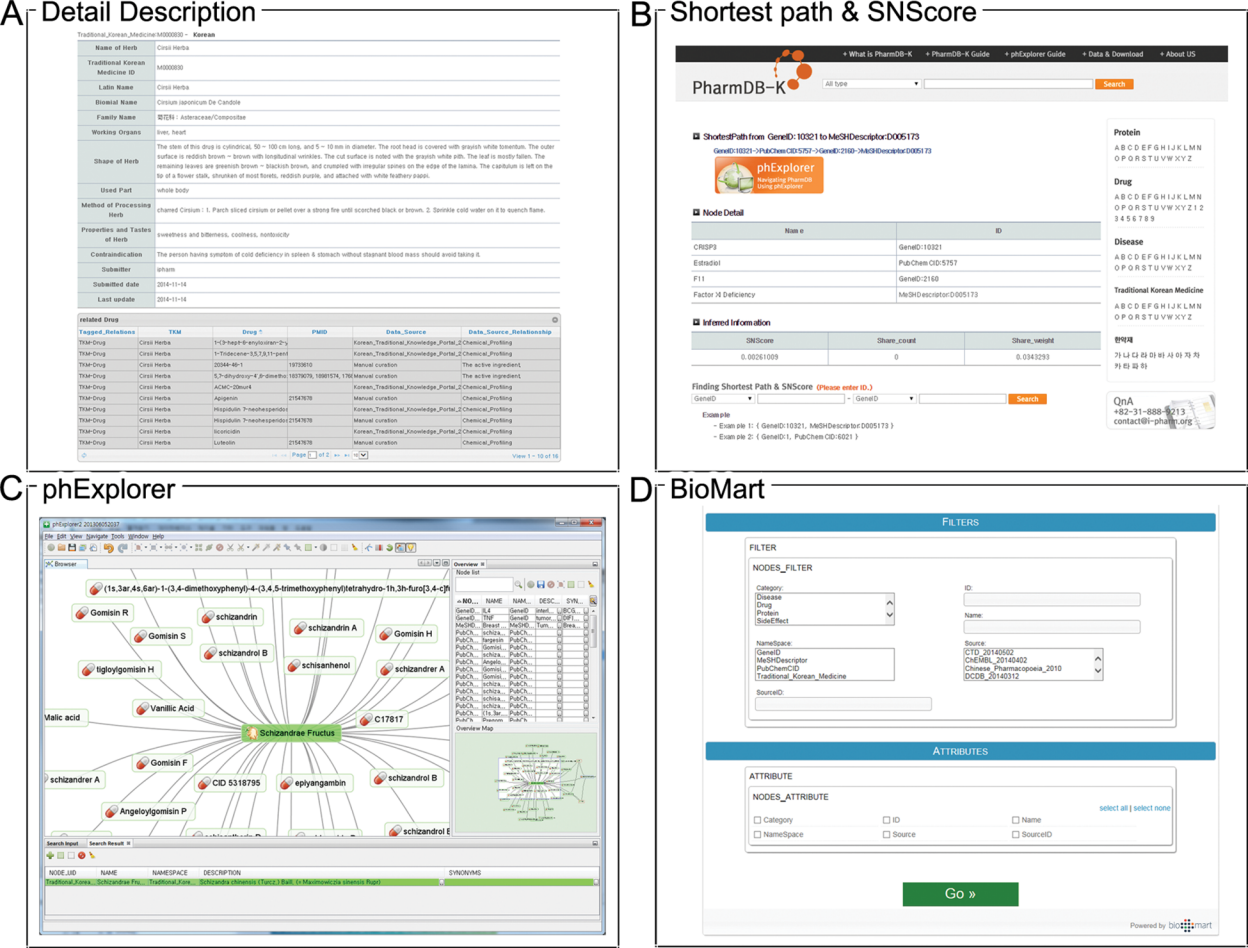
Web interface and tools. (A) Detailed information page. (B) Finding the Shortest Path. (C) phExplorer, a network visualization software for PharmDB-K. (D) PharmDB-K BioMart.

PharmDB-K resources are provided through a web interface (Fig 6A and 6B). PharmDB-K contains comprehensive synonym data for TKM, Drugs, Diseases, and Proteins to facilitate the search. In addition to general browsing, the option of finding the shortest path between two nodes is also available. Since the data in PharmDB-K form a highly complex network, it is neither appropriate nor informative to browse PharmDB-K in a text format. So, we are providing PharmDB-K information with two different tools, phExplorer (a network visualization software) and BioMart web service (Fig 6C and 6D) [42]. With phExplorer, users can easily browse PharmDB-K data in an interactive and dynamic manner. BioMart is a freely available data federation framework for large collaboration projects [42] and allows users to access disparate and distributed databases and to build their own analysis pipelines using a single user interface.

## Discussion

Although TKM provides new potentials of herbs that are not covered by TCM, TKM has not been well recognized worldwide and there is no well-known TKM database. Therefore, we developed an integrated bio-pharmacological TKM database called PharmDB-K. One of the most frequently stated challenges in the development of new TKM-based drugs is discovering active compounds and target proteins. An objective of PharmDB-K was to build a comprehensive bio-pharmacological network to explore the potential targets and indications for TKMs. PharmDB-K has several distinct advantages over existing TCM/TKM databases. By integrating bio-pharmacological databases, PharmDB-K provides 1) potential active compounds of TKM; 2) inferred links between TKM and potential target proteins. One of the most valuable information in PharmDB-K is the indicator compound information which collected from pharmacopoeias of five Asian countries. Since the indicator compound information is used to verify medicinal performance of herbs in each country, these compounds have great potential to be active compounds. Thus, PharmDB-K is able to suggest functional mechanisms of herbs from connections of the indicator compound information in TKM with known protein-disease network. This approach would be beneficial in accelerating TKM-based drug discovery. The other key information that PharmDB-K can predict is target proteins. Usually, researches on TKMs have focused on the activity measurements of some enzymes that have been known to be related to particular diseases because target proteins could not be postulated. To overcome this obstacle, PharmDB-K generated inferred TKM-Protein relationships using commonly shared compounds and diseases. It is based on the basic principle that the connection probability of a link between two different nodes is roughly proportional to the number of nodes commonly shared between them in bio-pharmacological network [41]. As shown in Fig 5, target proteins for TKMs were successfully predicted using inferred links. We believe that the systematic approach based on integrated bio-pharmacological network, such as PharmDB-K, is a promising way to uncover hidden TKM-Protein relationships and to expedite the elucidation of TKM-mediated mechanisms for a successful drug discovery. With manual curation, PharmDB-K offers more reliable and comprehensive compound and indication information for TKMs. Furthermore, phExplorer and BioMart will be very useful not only to researchers unfamiliar with databases, but also to bioinformaticians who want to carry out analyses using multiple databases. In conclusion, PharmDB-K has been designed to introduce TKM to the cutting edge drug discovery research field. We believe that PharmDB-K provides new insights on TKM-originated drug development research. We intend to continue efforts to expand our database by mining and analyzing published articles, and we plan to import prescription information in the near future to adopt combinatorial therapy concepts as well.

## Materials and Methods

### Cell viability assay

2,000 cells of MDA-MB-231 (purchased from ATCC) which are cultured in RPMI media containing 10% fetal bovine serum (FBS) and 1% antibiotics, were seeded in 96 well plates and incubated for 12 hr. Vanillic acid, L-Malic acid, Schizandrin and Syringin (Eleutheroside B) were purchased from Sigma. After 12 hr, they were dissolved in DMSO and treated in 5% FBS-containing media dose dependently. After 24, 48 and 72 hr, MTT reagent (5mg/ml, Sigma) was added to each well, and the plates were incubated in 37^°^C for 2 hr to check the cell viability. Purple-colored formazan dissolved in DMSO was analyzed spectrophotometrically at 570nm using ELISA plate reader. All the experiments were repeated three times.

### Western blot analysis

HaCaT cells were kindly gifted from Dr. Zigang Dong (Hormel Institute, University of Minnesota, MN, USA) and were maintained routinely in DMEM medium supplemented with 10% fetal bovine serum and a 100 ng/ml penicillin/streptomycin/fungizone mixture at 37°C in a humidified atmosphere of 5% CO2/95% air. Cells were incubated with TPA in the presence or absence of caffeic acid. After treatment, cell lysates were prepared according to the procedure described earlier [43]. The protein concentration was determined by using either the BCA or the BioRad protein assay kit. In some experiments, cytosolic and nuclear proteins were obtained from cells [43]. Protein samples (30–50 μg) were subjected Western blot analysis. Membranes were probed separately with antibodies against COX-2 (RB-9072-P1; Thermo Scientific, Rockford, IL), actin (sc-47778; Santa Cruz Biotechnology, CA, USA), IκBα (sc-847; Santa Cruz), p65 (clone D14E12, Cat No. 8242; Cell Signaling Technology, Beverly, MA, USA), Lamin B (sc-6216; Santa Cruz), and α-tubulin (sc-5286, Santa Cruz), and then blots were visualized according to the procedure described previously [43].

### Electrophoretic mobility gel shift assay (EMSA)

The EMSA for NF-κB DNA binding was performed using a DNA-protein binding detection kit, according to the manufacturer’s protocol (Gibco). The nuclear extract was prepared from cells incubated with TPA in the presence or absence of caffeic acid. The NF-kB oligonucleotide probe 5’-GAG GGG ATT CCC TTA-3’ was labeled with [γ-32P] ATP. Oligonucleotide probes containing NF-κB consensus sequences were obtained from Promega (Madison, WI, USA). The transcription factor-DNA binding assay was performed as described previously [43].

### Reverse-transcriptase polymerase chain reaction (RT-PCR)

RNA was isolated from HaCaT cells using TRIZOL® (Invitrogen). One μg of total RNA was reverse-transcribed with murine leukemia virus reverse transcriptase (Promega) at 42^°^C for 50 min and 72^°^C for 15 min. The cycling conditions were as follows: 5 min at 94^°^C followed by 27 cycles at 94^°^C, 1 min; 60^°^C, 1 min; 72^°^C, 1 min for il-8; 25 cycles at 95^°^C, 1 min; 70^°^C, 30 sec; 72^°^C, 1 min for gapdh followed by a final extension at 72^°^C for 10 min. Primer pairs (forward and reverse, respectively) were: il-8, 5’-ATGACTTCCAAGCTGGCCGTGGCT-3’ and 5’-TCTCAGCCCTCTTCAAAAACTTCT-3’; gapdh, 5’-GCATGGCCTTCCGTGTCCCC-3’ and 5’-CAATGCCAGCCCCAGCGTCA-3’.

### Cytokine ELISA

Murine Raw264.7 macrophage cells lines (purchased from ATCC) were cultured in DMEM (Gibco) supplemented with 10% fetal bovine serum (GenDepot), 100 U/ml penicillin (Gibco), 100 μg/ml streptomycin (Gibco), and 10 μg/ml gentamycin (Gibco). Cells were maintained at 37^°^C in a humidified atmosphere of 5% CO_2_. Raw264.7 cells were stimulated with 100 ng/ml of LPS (Sigma-Aldrich) in the presence of indicated concentration of Ginseng Radix extract, Angelicae Gigantis Radix extract, ginsenoside Rb1, decursin, or DMSO as a vehicle for 24 h for cytokine ELISA. The levels of IL-6 and TNF-α in the cultured supernatants were measured with ELISA kit (BioLegend). Antibodies used were anti-mouse IL-6 (MP5-20F3, Biolegend), Biotin-conjugated anti-mouse IL-6 (MP5-32C11, Biolegend), anti-mouse TNF-α (6B8, Biolegend), and Biotin-conjugated anti-mouse TNF-α (MP6-XT22, Biolegend). Assays were performed according to the manufacturer’s protocol. Data were analyzed with GraphPad Prism 6 software (GraphPad software). Statistical significance was calculated by two-tailed student’s t-test. Values of P < 0.05 were considered as statistically significant.

## Supporting Information

S1 Table (XLSX)
